# Drug therapy for patients with systolic heart failure after the PARADIGM-HF trial: in need of a new paradigm of LCZ696 implementation in clinical practice

**DOI:** 10.1186/s12916-015-0272-0

**Published:** 2015-02-18

**Authors:** Gerasimos Filippatos, Dimitrios Farmakis, John Parissis, John Lekakis

**Affiliations:** Heart Failure Unit, Department of Cardiology, Athens University Hospital “Attikon”, 1 Rimini St, 12462 Haidari, Athens, Greece

**Keywords:** Heart failure, Therapy, LCZ696, PARADIGM-HF, Angiotensin-converting enzyme inhibitor, Neprilysin, Valsartan, Sacubitril

## Abstract

Heart failure represents a primary cause of morbidity and mortality in older people and despite significant therapeutic advances, it is still characterized by important unmet needs, thus remaining a challenging field of clinical research. The recent PARADIGM-HF trial compared the novel compound LCZ696, a combination of the angiotensin receptor blocker valsartan and the neprilysin inhibitor sacubitril, versus the angiotensin-converting enzyme inhibitor enalapril in 8,442 patients with symptomatic chronic systolic heart failure. LCZ696 led to a 20% reduction in the rate of death or hospitalization for heart failure and a 16% reduction in the rate of all-cause death compared to enalapril at 3.5 years of follow-up. Despite those impressive results, the clinical application of this novel agent that requires the substitution of a cornerstone of current heart failure therapy, the angiotensin-converting enzyme inhibitors, should follow careful steps as imposed by the study design, the recruited population and the outcome in specific patient subgroups. Further insights into the effects of LCZ696 will be provided by the ongoing PARAGON-HF trial in patients with diastolic heart failure.

## Background

Heart failure (HF) represents a main cause of morbidity and mortality and the first reason for hospital admission in older people [1]. The important advances in the treatment of HF accomplished over the past decades in terms of drug and device therapy have resulted in a significant improvement in the prognosis in patients with chronic HF. The cornerstone of modern drug therapy in chronic HF is the inhibition of neurohormonal activation that plays a crucial role in the pathophysiology of HF development and progression and, more specifically, of the renin-angiotensin-aldosterone system and the sympathetic nervous system [2]. Accordingly, all patients with HF with reduced ejection fraction, unless having a contraindication, should receive an angiotensin converting enzyme inhibitor (ACEi) or an angiotensin receptor blocker (ARB) if non tolerant to ACEi, plus a beta-blocker with the further addition of a mineralocorticoid receptor antagonist (MRA, aldosterone or eplerenone) if still symptomatic [2].

The incidence of HF keeps rising owing mainly to the aging of the population and the effective management of formerly lethal conditions, such as acute coronary syndromes. This is followed by an increasing trend in HF hospitalizations along with an enormous economic burden that also keeps growing [3,4]. In addition, the outcome of patients hospitalized for an acute HF episode remains quite poor, with unacceptably high post-discharge mortality and re-hospitalization rates [1]. Finally, although drug and device therapy has been proven beneficial for patients with systolic HF, currently termed HF with reduced ejection fraction (HFrEF), the same is not true for those with diastolic HF or HF with preserved ejection fraction (HFpEF), who roughly represent half of the total HF population and in whom no evidence-based therapy is yet defined [2]. Therefore, HF represents a growing medical, public health and financial problem with several unmet needs. In this context, HF remains a challenging field for the search for novel therapeutic agents that would further improve patients’ outcomes.

### A PARADIGM shift in heart failure therapy

The recently published ‘**P**rospective comparison of **A**ngiotensin **R**eceptor neprilysin inhibitors with **A**ngiotensin converting enzyme inhibitors to **D**etermine **I**mpact on **G**lobal **M**ortality and morbidity in **H**eart **F**ailure’ (PARADIGM-HF, NCT01035255) trial applied an innovative approach to the introduction of new HF therapies. Instead of adding the new agent on top of standard care, an approach followed by the majority of previous HF clinical trials [2,5], PARADIGM-HF proposed the substitution of one of the cornerstones of modern HF therapy, the ACEi [6]. In addition, PARADIGM-HF focused on the inhibition of the endopeptidase pathway, an import component of the renin-angiotensin-aldosterone system not addressed by the current HF therapies.

The new compound with the code name LCZ696 (400 mg daily), a combination of the ARB valsartan and the neprilysin inhibitor sacubitril, was compared with the ACEi enalapril (20 mg daily) in 8,442 patients with symptomatic chronic HFrEF and increased levels of natriuretic peptides [5]. At 3.5 years of follow-up, LCZ696 had led to a 20% reduction in the incidence rate of death or HF hospitalization and a 16% reduction in the incidence rate of all-cause death compared to enalapril, results that were highly statistically significant. Regarding safety, LCZ696 was followed by lower rates of hyperkalemia, renal dysfunction and cough but higher rates of hypotension. According to a secondary analysis, LCZ696 prevented the clinical deterioration or progression of surviving patients in terms of required treatment intensification of therapy, hospital visits or admissions and use of advanced management modalities (inotropes, assist devices, transplantation) more effectively than did enalapril [7]. Thus, the question that inevitably arises is whether keeping HF patients on ACEi is still ethical given those remarkable results.

### The challenge of clinical application

If one takes a better look at the design of the PARADIGM-HF trial, it seems that the clinical application of LCZ696 needs to follow careful steps. First, the study was preceded by a single-blinded run-in phase, during which about 10% of patients dropped out mainly due to adverse events or abnormal laboratory results [5]. Although a run-in phase enhances a trial’s internal validity by limiting treatment discontinuation, on the other hand it limits the inference of the results to the general HF population, thus affecting the external validity. In addition, two short washout periods of 36 hours were applied between enalapril and LCZ696 run-in periods and at the end of the run-in phase to avoid overlapping of ACEi and LCZ696 that would increase the risk of angioedema, a fact that renders inference of the trial even more complicated.

According to the trial’s subgroup analyses, LCZ696 was ‘less’ beneficial in ACEi-naïve patients, while LCZ696 led to a higher incidence of symptomatic hypotension compared to enalapril, which, however, did not result in a higher treatment discontinuation [5,6,8]. Those concerns are particularly important in patients hospitalized for acute HF, in whom the use of oral medication including angiotensin aldosterone system inhibitors is not sufficiently guided by available evidence [9]. Furthermore, patients who are on enalapril or equivalent ACEi doses higher than those studied in the trial or those treated with angiotensin receptor blockers may also represent a challenge as they have not been included in PARADIGM-HF. Finally, the cost-effectiveness of this new strategy remains to be assessed. The HF patient subgroups that may pose a challenge for the clinical application of LCZ696 according to the aforementioned issues are outlined in Table 1.

**Table 1:** **Specific heart failure patient subgroups representing a challenge for the implementation of LCZ696 in clinical practice**

Clinical characteristics	Marginally low blood pressure
	Hospitalization for AHF
	NYHA IV class
	Advanced heart failure
Drug therapy-related characteristics	ACEi-naïve patients
	Intolerance to ACEi or ARB
	Low ACEi dose
	High ACEi dose
	Patients on ARBs therapy

ACEi, angiotensin converting enzyme inhibitors; ARB, angiotensin receptor blockers; NYHA, New York Heart Association.

## Conclusions

The results reported by the PARADIGM-HF trial investigators are indeed impressive and introduce a paradigm shift in the treatment of chronic HFrEF. However, the clinical application of the novel agent LCZ696 will definitely require a new paradigm for many HF patients. Further to PARADIGM-HF, the ongoing PARAGON-HF trial addresses the effects of LCZ696 on patients with HFpEF, a condition characterized by a total lack of evidence-based therapies, as stressed earlier, thus leading to high expectations for another major paradigm shift in HF.
